# Data Resource Profile: the Scottish Social Care Survey (SCS) and the Scottish Care Home Census (SCHC)

**DOI:** 10.23889/ijpds.v4i1.1108

**Published:** 2019-09-02

**Authors:** D Henderson, JK Burton, E Lynch, D Clark, J Rintoul, N Bailey

**Affiliations:** 1 Urban Big Data Centre, University of Glasgow, Glasgow, G12 8RZ; 2 Academic Geriatric Medicine, Institute of Cardiovascular and Medical Sciences, University of Glasgow, Glasgow, G12 8TA; 3 Health and Social Care Analysis Division, Scottish Government, Edinburgh, EH1 3DG; 4 Indexing Team, National Records of Scotland, Edinburgh, EH12 7UT

## Abstract

**Introduction:**

Linked health care datasets have been used effectively in Scotland for some time. Use of social care data has been much more limited, partly because responsibility for these services is distributed across multiple local authorities. However, there are substantial interactions between health and social care (also known internationally as long-term care) services, and keen policy interest in better understanding these. We introduce two social care resources that can now be linked to health datasets at a population level across Scotland to study these interdependencies. These data emerge from the Scottish Government’s centralised collation of data from mandatory returns provided by local authorities and care homes.

**Methods:**

Deterministic and Probabilistic methods were used to match the Social Care Survey (SCS) and Scottish Care Home Census (SCHC) to the Community Health Index (CHI) number via the National Records of Scotland (NRS) Research Indexing Spine.

**Results:**

For the years 2010/11 to 2015/16, an overall match rate of 91.2% was achieved for the SCS to CHI from 31 of Scotland’s 32 local authority areas. This rate varied from 76.7% to 98.5% for local authority areas. A match rate of 89.8% to CHI was achieved for the SCHC in years 2012/13 to 2015/16 but only 52.5% for the years 2010/11 to 2011/12.

**Conclusion:**

Indexing of the SCS and SCHC to CHI offers a new and rich resource of data for health and social care research.

## Background

In common with other Western nations, Scotland is challenged by the most effective ways to support increasing numbers of adults with complex health and social care needs as a consequence of rising levels of multimorbidity and frailty, associated with population ageing [1, 2] . Identifying ways to deliver good quality and efficient care is of high importance for all industrialised nations but particularly those, such as the UK, that have chosen to have a prolonged period of constraint on public spending or ‘austerity’ [3].

Linked health datasets have been effectively used in Scotland for research and evaluation for some time. The foundations for this lie in standardised recording of medical diagnoses and treatments, requirements to report or share data with the national intelligence hub for the Scottish health service, and a ready means of linkage to the national population spine through the health service identification number, the Community Health Index (CHI) [4].

By contrast, access to social care data at a national level has been very limited [5]. A particular challenge is that social care is a responsibility of 32 individual local authorities which collate and store data in their own ways. From 2010, however, the Scottish Government began to require all local authorities to make annual returns in a consistent format. Not only did this provide a national picture for social care, it also raised the prospect of linkage to the national population spine which would in turn enable linkage to health care and other records. Further barriers to use of the data include a need for joint working to understand the organisational cultures and practices which sit beneath non-health data collections as well as skills needed to work with this kind of complex, messy administrative data[6, 7].

New impetus for cross-sectoral data linkage has been provided by efforts to achieve greater service integration between health and social care. Since April 2016, the planning and provision of these services have been legally integrated in Scotland, explicitly acknowledging the interactions between them. This has led to significant reorganisation in operational structures. Integration Authorities (IAs) have been formed to control the budgets previously held separately by NHS Health Boards and local authorities [8]. Accurate, population-wide, routinely-collected health and social care data sources are critical to ensuring these changes deliver on the intended improvements in efficiency and effectiveness [9, 10].

The aim of this article is to enable and encourage use of these resources by providing an overview of the two national social care data sources, the Social Care Survey and the Scottish Care Home Census, and by summarising the work to make them linkable to health and other administrative data. Brief summaries of each resource are provided, beginning with the Social Care Survey. These include: an overview, description of data collection, variables, data quality, strengths, and limitations. We also outline the procedures for obtaining access to the data which are common to both resources. Finally, we describe the indexing process used to match these resources to the population spine, which enables cross-sectoral linkage.

## Social Care Survey

### Overview

The Social Care Survey (SCS) is collected by the Scottish Government’s Health and Social Care Analysis Division (HSCAD) from each of the 32 Scottish Local Authorities. The survey is used to produce an annual report of aggregate statistics detailing the types and amount of social care provided by each local authority [11]. Individual-level data have been collected since 2010, initially in two separate surveys. These were merged in 2013 as the SCS. Certain measures captured by the SCS are used in funding formulae to calculate allocation of resources to each local authority (e.g. number of people receiving home care) [12]. A description of key terms related to the SCS is provided in table 1.

**Table 1: Key terms relating to SCS:** 

Term	Description	No. of individuals recorded as receiving service in SCS 2016/17
Community alarm	A technology package which consists of a communication hub plus a button/pull chord/pendant which transfers an alert to a monitoring centre or individual responder.	128,750 (alarm and telecare services counted together)
Telecare	Remote or enhanced delivery of care of services to people in their own home by means of telecommunications and computerised services. E.g. linked pill dispensers, linked smoke detectors etc.	
Home care	Practical services (excluding 24-7 care) which assist individuals to function as independently as possible and/or continue to live in their own home, e.g. Routine household tasks (basic housework, shopping, laundry, paying bills), personal care (e.g. personal hygiene, continence management), respite care in support of the individual’s regular carers, or care provided to individuals living in sheltered housing or supported accommodation.	59,640
Meals	Provision and delivery of either hot or frozen meals to individuals. Often described as “Meals on wheels”.	6,390 (likely to be an underestimate – poor data collection)
Housing Support	Housing support services help people manage their home in different ways. These include assistance to claim welfare benefits, fill in forms, manage a household budget, keep safe and secure, get help from other specialist services, obtain furniture and furnishings and help with shopping and housework. The type of support that is provided will aim to meet the specific needs of the individual	18,940 (likely to be an underestimate, poor data collection)
Self-Directed Support	SDS gives people control over an individual budget and allows them to choose how that money is spent on the support and services they need to meet their agreed health and social care outcomes. The 4 options are: 1. Cash payment 2. Allocation of budget to a provider chosen by the individual 3. Individual chooses to allow the local authority to arrange care 4. A mixture of options 1,2, or 3 for different types of support.	83,770
Free Personal Care	Help with personal care (such as personal hygiene, continence management, food & diet, problems with immobility, counselling & support, simple treatments, or personal assistance) provided by Scottish Government free to those assessed by their local authority as needing it.	Not reported in SCS
IoRN score	The Indicator of Relative Need (IoRN) is a questionnaire consisting of 12 multiple choice questions divided into 5 sections: Activities of Daily Living, Personal Care, Food/Drink preparation, Mental well-being and behaviour, and bowel management. It provides a standardised means of grouping individuals according to their relative needs.	Not reported in SCS

Data is initially collected by local authorities as part of their social care management systems with requested SCS variables submitted to HSCAD via a secure web-based system called ProcXed. This system includes integrated validation checks and facilitates speedy information exchange.

### Frequency of data collection

Data is obtained annually using different time periods for different parts of the service. Data relating to individuals receiving home care, meals, or housing support is reported for a single census week while data on those receiving community alarms, telecare or self-directed support is reported for the whole financial year. The census week usually includes the date 31st March whilst the financial year is defined as 1st April to 31st March [13].

Prior to 2013, telecare and community alarm data variables were also collected for the census week only as opposed to the extended financial year used in more recent years. This creates a discontinuity in the data which needs to be borne in mind for comparisons of trends.

### Variables

Table 2 presents the variables collected by the SCS . Each of these variables is available for the surveys 2010/11 to 2015/16, with the exception of variables relating to Self-Directed support. These are available from 2014/15 onwards reflecting the introduction of this policy in April 2014.

**Table 2: Summary of variables available from SCS data:** 

Variable Name	Definition/Explanation
**Demographic variables**	

Local Authority	Name of Local Authority providing care
Postcode	Postal area code
Datazone	Scottish neighbourhood statistics small area geography datazone relating to postcode of client
Date of Birth	
Age	
Gender	Multiple choice. 1. Male 2. Female
Ethnic Group	Multiple choice – 6 potential levels. 1. White, 2. Mixed or multiple ethnic groups 3. Asian, Asian Scottish or Asian British 4. African, Caribbean or Black 5. Other 6. Ethnic Background Not Disclosed
Alone	Flag indicating whether client lives alone
Type of Housing	Multiple choice 1. Mainstream 2. Supported 3. Long-stay Care Home 4. Hospital or another medical establishment
Carer	Flag indicating whether client is known to have a carer
Care Plan Date	Date of care plan review (optional)
IoRN Date	Date Indicator of Relative Need assessment (optional)
IoRN score	Indicator of Relative Need score (optional)
Housing Support	Flag indicating whether client received housing support
Hot Meal	Flag indicating whether client received Meals on Wheels (Hot)
Frozen Meal	Flag indicating whether client received Meals on Wheels (Frozen)
Home Care	Flag indicating whether client received Home Care
Social Work	Flag indicating whether client is known to have a social worker or support worker
Alarm	Flag indicating whether client received a community alarm
Telecare	Flag indicating whether client received another form of telecare other than an alarm
SDS	Flag indicating whether client received Self-directed Support funding

**Client Group Variables**	

Client Group	Group that client belongs to (more than one category can be returned) – 9 potential levels: Dementia, Mental Health Problems, Learning Disability, Physical Disability, Addiction, Palliative Care, Carer’s, Problems arising due to Infirmity due to age, Other.

**Home Care Variables**	

Total weekly home care hours	Scheduled number of weekly home care hours provided, available split by provision (e.g. Local Authority, Private provider etc.), scheduled or actual hours, and number of Free Personal Care hours provided
MultiStaff	Indicated if two or more staff are required for home care visits

**Self-Directed Support Variables**	

SDS Option	Whether receiving option 1, 2, or 3 (Multiple options can be returned)
SDS start date	Start date of time period associated with SDS Care Package
SDS end date	End date of time period associated with SDS Care Package
TotValueSDS	Total Value of the SDS package
ValueSDS1	Total Value of SDS1 package
ValueSDS2	Total Value of SDS2 package
ValueSDS3	Total Value of SDS3 package (optional)
SDSContrib	Contributors to Financial Value of Total Care Package – 7 potential levels: Social Work, Housing, Independent Living, Health, Client, Other, Not known
SDSNeeds	Type of assessed support needs provided through SDS – 10 potential levels: Personal Care, Health Care, Domestic Tasks, Housing Support, Social Educational Recreational, Equipment & Temporary Adaptions, Short Breaks, Meals, Other, Not known
SDSSupport	Type of support mechanism provided through SDS – 6 potential levels: Client employs one/more Personal Assistant, Client purchases service from or has service provided by Local Authority, Client purchases services from a private provider, Client purchases services from a voluntary provider, Client purchases services from another source, Not known.

### Quality and completeness of data

The method of data collection used for the SCS, particularly the census week variables, means that it does not capture every individual who receives social care in any financial year. The proportion of social care recipients captured by the survey is unknown, although some work attempting to quantify this is underway [14]. There is little reason to suspect collection of census data at the end of March results in an unrepresentative sample in any way. However, until a formal assessment of the representativeness of the survey is available, caution is required when reporting results.

In terms of useable variables for research purposes, HSCAD’s own reporting of data quality [13] reveals data on meal services is poorly completed and does not allow for accurate assessment of national coverage. Furthermore, in addition to under-reporting of those with dementia in the “Client Grouping” variable, there is also ambiguity across local authorities in how frail, older people and those with physical disabilities are classified. The implementation of the Self-Directed Support Act (2014) has led to large discrepancies in the way SDS variables were recorded by local authorities. Coupled with varying levels of missing data these variables appear unsuitable for research purposes.

**Figure 1:**
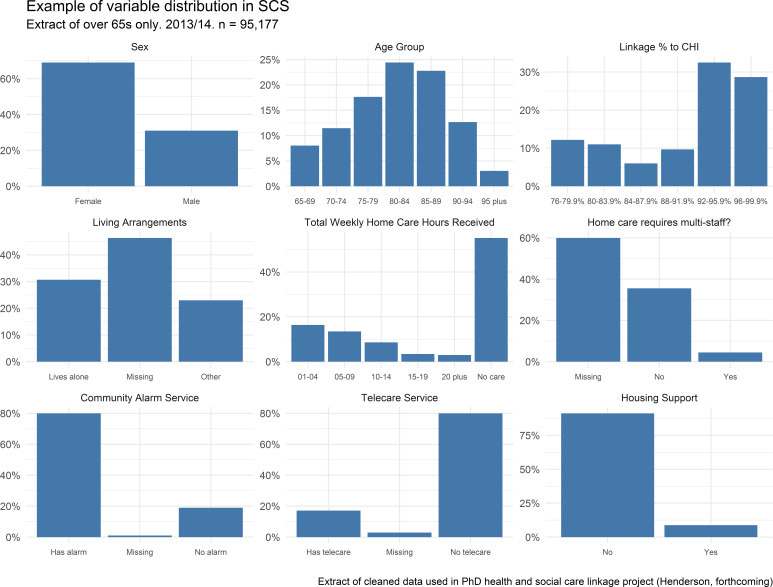


The distribution of variable values in 2014/15 used in an extract of the SCS linked to health data for a PhD thesis [15] is shown in figure 1. Disappointingly, given its potentially high research use, the Indicator of Relative Need (IoRN) (see table 1) variable has ~90% records with missing data and was therefore omitted in the data cleaning process. The Type of Housing variable was also omitted for similar reasons. This extract includes only those over the age of 65 – approximately three-quarters of all social care users (n=95,177). As the extract was part of a linked data project, age and sex variables were derived centrally from the CHI population spine and therefore do not reflect actual SCS collection. Linkage rate to CHI varied by local authority (described more fully below) and figure 1 shows almost 60% of individuals in the extract lived in a local authority with a linkage rate above 92%. Living arrangements and multi-staff variables have high levels of missing data with sub analyses showing variation at the local authority level. This suggests data are missing-not-at-random discounting the use of imputation methods. Unsurprisingly, as numbers of those receiving home care are used to calculate allocation of funds to each local authority [12]these figures are well returned as are those detailing community alarm and telecare services (all variables of significant interest for policy makers).

### Strengths

As the Scottish Government is the sole data controller for the SCS covering years 2009/10 to 2016/17, permission to use the data can be granted from this single source rather than from each individual local authority. This means the SCS provides a standardised, individual-level source of social care provision with national coverage. This is unique in terms of routine data available in the United Kingdom.

Recent indexing of the SCS to the CHI number via the National Records of Scotland (NRS) Research Indexing Spine (described below) offers the opportunity to link this data, at a population level, to health datasets which are also indexed with a CHI number. Given recent structural changes in the delivery of health and social care services in Scotland and the wider UK, data linkage can enable analysis of how these services interact, how they are accessed across the country, and measure the impact of social care services on a range of health-based outcomes.

### Limitations

As described below, indexing to the CHI register had good overall match rates of 91.2%. However, these rates vary across local authority areas (from 76.7% - 98.5%) meaning caution is required when comparisons are made across council areas. Whilst it is likely that the SCS captures many of those receiving social care in a financial year, the exact proportion is unknown. This means estimating the total number of social care recipients is impossible. Finally, potentially valuable variables (e.g. living arrangements indicating whether an individual lived alone, their client grouping, and relative need scores) suffer from high levels of missing data and/or data quality issues.

### Usage to date

Indexing of the SCS to the CHI central register was completed in Summer 2017. At present several Scottish Government/ESRC-funded PhD projects have applied for and been granted access to link the SCS to various health datasets with results pending. These include projects aiming to identify the relationships between multimorbidity and social care [15] and to ascertain the influence of unpaid carers for those receiving personal care services [16]. No academic studies have been completed with the SCS as a stand-alone resource.

## Scottish Care Home Census

### Overview

The Scottish Care Home Census (SCHC) is collected by the Scottish Government Health and Social Care Analysis Division and the Care Inspectorate. The Care Inspectorate is the national regulator of care services in Scotland and it is mandatory for all care home services to register with them. The data are collated and quality assured by the NHS National Services Scotland Information Services Division (NHS NSS ISD) who produce a summary publication of key findings.

The SCHC dataset includes information provided by the Care Inspectorate about all care home services registered that year, supplemented by data completed by care home staff about the home and their residents. Submission is not mandatory, but actively encouraged for all adult care home services. The census submission is split into two parts, the first requests aggregate data about the care home and the second collects individual data about residents. Care home admissions are categorised into those for short-stay, respite or long-stay. Only those classified as long-stay residents are included in the individual resident data collection. Long-stay residents are defined as those whose intention at admission was to be a permanent resident, whereas short stay residents are those remaining for less than six weeks [17].

Individual resident data from 2010/11 to 2015/2016 have been indexed to allow records from the SCHC to be linked to other national datasets using the Scottish individual identifier variable the Community Health Index (CHI) number. The indexing methodology is described in full below.

### Frequency of data collection

Data are collected as an annual submission per financial year (1st April – 31st March). However, the request is for information on care home activity and residents throughout the year. Data collection started in 2003, moving to an electronic format in 2010. The e-form is open from July to May to allow homes to enter data on their residents contemporaneously.

### Variables

Table 3 presents the aggregate and individual variables which are available from the SCHC data on an annual basis. A full description of the Scottish care home population, and distribution of variables, based on four years of SCHC data has been provided elsewhere [18].

**Table 3: Summary of aggregate and individual variables available from the SCHC data:** 

Aggregate variables	

*Variable name*	*Definition/explanation*
Subtype (of care)	Older people; learning disabilities; mental health problems; physical & sensory impairment; physical disabilities
Service type (provision)	Private; local authority; voluntary or not for profit; health board
Local authority area	Location of care home
Health board area	Location of care home
Capacity	Registered total number of beds
Main provision	Main client group for whom the care home can provide care, entered as part of the annual return to the Care Inspectorate
Client needs	Seventeen categories of ‘client need’ from which the care homes are asked to select all those relevant, entered as part of the annual return to the Care Inspectorate. Categories include: Alcohol, alcohol brain injury, acquired brain injury, autism, blood borne viruses, dementia, drug dependency, Korsakoffs, learning disabilities, mental health (not dementia), mothers and children, older people, older people (dementia), older people (frailty), palliative care, physical disability or illness, visual impairment
Weekly charges	Includes charge for those self-funding versus local authority funding and those with and without nursing care
No of admissions	Totals requested for short stay, long stay and respite care
No of discharges	Totals requested for short stay, long stay and respite care
No of deaths	Totals requested for short stay, long stay and respite care
Residents (at census)	Totals requested for short stay, long stay and respite care

Individual long-stay resident variables	

*Variable name*	*Definition/explanation*

Date of birth	Month/year
Sex	Female, Male, Other
Ethnicity	White, Other Ethnic Group; Not Disclosed; Not Known
Date of admission	Month/year
Source of admission	Another care home, hospital, other/not known, own home, and sheltered/supported accommodation
Date of discharge	Month/year
Reason for discharge	Another care home, death, hospital, other/not known, own home, and sheltered/supported accommodation
Mainly or wholly funded	Source of funding for placement providing majority or all of the costs
Free personal care & free nursing care	If individuals are privately funding their care, are they receiving the free personal care allowance and the free nursing care allowance
Local authority funding placement	If individuals are funded by their local authority, which one funds their placement
Clinical variables	Requires nursing care; dementia medically diagnosed; dementia not medically diagnosed; visual impairment; hearing impairment; acquired brain injury; learning disability; other physical disability or chronic illness; mental health problems; alcohol dependency; drug dependency; none of these

### Completeness of data

One challenge is to obtain an accurate denominator using the dataset. The Care Inspectorate change the service identification number when a care home changes its registration with them. Some of these changes reflect genuine differences, e.g. closure of care home service, but many involve smaller administrative changes while the building and residents remain unchanged. This makes it difficult to establish how many services were active at any specific point in time and thus to quantify how complete the data are.

### Strengths

The care home population is difficult to identify within UK routine data sources [19, 20], thus the existence of a national data source offers significant potential. Making the SCHC linkable to other routine data sources provides opportunities to enhance the information collected within the census to help understand a difficult to research population [21]. If the insights offered by analysing the data are useful, use of the SCHC data collection infrastructure could provide a platform for improving and enhancing the data collected.

One of the key strengths of the SCHC data are their inclusivity. All sectors of care are invited to participate and are included, meaning private, not-for-profit and local authority care homes are included. This is in contrast to usual approaches which have been provider-led [22] or only include those funded by their local authority [23]. The SCHC also includes data on all adult care home services, without restrictions around age and service type. This is important as, although the majority of care home residents are older adults, younger age groups are a significant group, often with complex needs, who can otherwise be overlooked.

The data are collected by care home staff, using a standardised electronic data collection form and can be done throughout the year, rather than relying on an annual snapshot count.

### Limitations

As data collection is not mandatory, the SCHC does not capture data from all care homes and all long-stay residents in Scotland. This can introduce bias into analyses conducted using the data. It is difficult to estimate the impact of such bias as reasons for non-participation have not been explored.

There is no formal quality assurance process in place around the aggregate and individual data collection. The electronic form can place limitations, e.g. on count variables being totalled correctly, but there is no mechanism for evaluating the data submitted.

The resident dataset does not contain a resident identifier variable which is consistently applied between years. As such, use of the unlinked data is more limited as the same individuals are likely to be present in multiple years, but cannot be identified.

Finally, the range of variables collected on individual residents is limited in scope, lacking any resident experience measures and important clinical measures such as frailty, medication use, dependency.

### Usage to date

The SCHC data have been included as one of several care home data sources to ascertain care home admission as a clinical trial outcome. In this project, two randomised trial cohorts were linked to care home data sources to investigate the association between statin use and the need for institutional care [20]. The primary purpose of linking the SCHC data was to explore pathways into care from hospital, compared to those who move from the community. Work on this linkage project is ongoing. While linkage was performed, some analysis has been conducted on the unlinked SCHC data to provide descriptive epidemiology of the population.

## Data resource access

Researchers wishing to use the SCS or SCHC data require permission from the Scottish Government as the data controller via HSCAD (https://www2.gov.scot/Topics/Statistics/About/DataAccess/DAPanel). If they wish to use the SCS or SCHC data linked to other data sources, they must also apply to the Public Benefit and Privacy Panel (PBPP) for Scotland [24], via the electronic Data Research and Innovation Service (eDRIS) infrastructure. PBPP applications require completion of an application form and a complete list of variables requested for the project. Essential to this process is the demonstration of how proposed research has a public benefit and the measures taken to ensure the privacy of individuals to whom the data refer.

Those interested in only aggregate-level data can access either the annual SCS publication from the Scottish Government website [11] or the annual SCHC publication from the Information Services Division (ISD) website [25].

## Indexing to CHI

One of the key requirements for linkage of data between multiple sources is the presence of a variable which identifies each person in the data source as unique. This variable is generated by those performing the linkage, before personal identifying variables are removed, and the data are released to researchers in a secure environment. Individuals registered with a General Practitioner in Scotland have a unique identifier variable, known as the Community Health Index (CHI) Number. The ten-digit CHI includes an individual’s date of birth (DDMMYY) followed by a three-digit sequence and a check digit. This tenth value is always even for females and odd for males and is therefore unique for an individual [26]. The CHI itself is a population register and contains additional data about the individual which can be linked to other data sources [27]. Datasets which record CHI can therefore be more readily matched together using deterministic linkage, with greater certainty that the records all belong to the same individual [28].

From their inception, neither the Social Care Survey nor the Scottish Care Home Census recorded the CHI numbers. Therefore, they needed to undergo indexing to assign the CHI number based on the available personal identifiers. The indexing was performed by the NRS Indexing Team, independent from the researchers requesting access to the datasets. Reports and spreadsheets outlining the linkage process are provided as supplementary material.

### Methodology

The National Records of Scotland Indexing Team use a Research Indexing Spine, based on the NHS Central Register (NHSCR), which contains names, dates of birth, gender as well as historic postcodes for ~9 million individuals (including births, GP registrations and deceased individuals). The NHSCR is linked operationally to the CHI database managed by NHS Scotland, and a lookup between the Spine Identifier and CHI Number is held by the Indexing Team to facilitate linkage to health datasets for research purposes. They use both deterministic and probabilistic methods to match records to the Indexing Spine and produce reports on the linkage quality and potential biases associated with the linkage. This work is done separately from any clinical information contained within the datasets, which are not required to facilitate the indexing process. Datasets which do not contain names are linked using an approach originally developed in ISD to link school pupil census data managed by the Scottish Government ScotXed team to the CHI database. This probabilistic approach achieved up to a 98.5% success rate [29, 30] and NRS have since further developed this methodology to report match rates of the school census to CHI numbers of 99.0%.

### Indexing Social Care Survey (2010-2016)

Considerable variation in the quality of personal identifiable information used for indexing was found across local authorities. 29.6% of SCS records originating from 17 local authorities had missing values for day of birth or returned a value of “01” for most of their dates of birth. Records from these authorities were indexed separately using only month and year of birth (in addition to postcode and gender) for matching. The remaining local authorities could be matched normally using full dates of birth, gender and postcode. Using this method an overall linkage rate of 91.2% to CHI was achieved for 31 of the 32 local authorities. One local authority provided only partial dates of birth and postcodes resulting in very poor match rates. This necessitated removal of all records from that council area. The match rate for the remainder varied from 76.7% to 98.5% across council areas with 22 councils having a match rate over 90%. Assessment of potential biases found no discernible difference across ages, sex, or SIMD (deprivation) decile of residence.

### Indexing Scottish Care Home Census (2010/11-2011/12)

The early years of the SCHC data had a limited range of identifiers including care home postcode, sex and date of birth. Only 57.9% of 71,641 records had a valid date of birth, plus sex and postcode. Where all these identifiers were present, 90.9% could be linked to the Spine. However, due to the extent of missing data only 52.5% (37,579 records) could be matched to a single CHI number based on the available data.

### Indexing Scottish Care Home Census (2012/13-2015/16)

The later years of the SCHC also requested forename and surname in addition to gender, postcode, day, month and year of birth. The completeness of each of these fields varied from 88-100%. In total, 146,152 records were submitted for indexing. The indexing was undertaken in stages, including pre-processing of the data to use Soundex codes to improve surname and forename matching. This was followed by use of BigMatch linkage software, developed in the US Bureau of Census [31]. BigMatch allows data to be blocked to identify best matching pairs between the data source and the Indexing spine. Weights are then assigned based on the match probability. This means that records which have multiple identifiers are more readily matched than those with fewer, to try to reduce the likelihood of matching records which are not from the same individual. Deduplication was performed to ensure each individual CensusID is only assigned to a single individual, albeit they may have multiple census records as they appear in several years. Clerical review was conducted to evaluate the quality of the linkage, by comparing samples of the original records with the results of the index match. This review resulted in estimated precision for the indexing exercise of 98.6% (95%CI: 98.1-99.2%). Once indexing was complete, 131,229 records matched to a CHI record (89.8% of those submitted), representing 63,356 unique individuals. Assessment of potential biases found no discernible difference across ages, sex, or SIMD (deprivation) decile of residence.

## Conclusions

International datasets have allowed exploration of the role of social care services [32, 33] an area which has been under-researched in the UK context. This article provides an overview of Scotland’s two key Social Care datasets – the Social Care Survey and Care Home Census. It is designed to highlight the potential of these resources as tools to explore current practice, and inform relevant applied research designed to develop evidence-based policy. Potential improvements that could be made to enhance the quality of data available include: more robust collection strategies for person identifying information (PII) enabling more accurate linkage to CHI, longer census periods to capture a greater proportion of social care users, ensuring data collection is updated with stakeholder involvement to ensure we capture the changing role care homes are providing (e.g. intermediate care and respite usage) and record measures which can help understand the care home population and their needs at a population level. A more comprehensive understanding of this area using real, routinely collected data could help support the case for adequate investment in social care services to improve lives [34].

## Supplementary Files

Social Care Survey - read-through indexing

Social Care Survey, NRS Indexing Spine Linkage

Care Home Census 2010/11 - 2011/12 Indexing

Care Home Census 2012/13 - 2015/16 Indexing

## Abbreviations

CHI – Community Health Index

eDRIS – electronic Data Research and Innovation Service

ISD – Information Services Division

NHS NSS – National Health Service National Services Scotland

PBPP – Public Benefit and Privacy Panel

SCHC – Scottish Care Home Census

SCS – Social Care Survey

IoRN – Indicator of Relative Need
