# Supplementary material for: Data Resource Profile: the Scottish Social Care Survey (SCS) and the Scottish Care Home Census (SCHC)
Source: Int J Popul Data Sci. 2019 Sep 2;4(1):1108. doi: 10.23889/ijpds.v4i1.1108 (PMC8142955; doi:10.23889/ijpds.v4i1.1108)
Supplement: Social Care Survey - read-through indexing [file ijpds-04-1108-s001.pdf]

Social Care Survey - read-through indexing

Linkage Summary Results

Number of records input for Spine Matching		
- to be processed as normal using full DOB (CAs deemed good or DYOB > 1)	418,732	70.4%
- to be processed separately ignoring day of birth (Imperfect CAs and DYOB=1 or missing)	175,648	29.6%
- total records processed	594,380	100.0%
Number of Unique PersonIDs by category of input record from best matching record to Spine		
- full DOB records	377,171	77.3%
- partial DOB records	111,014	22.7%
- total unique PersonIDs	488,185	100.0%

Match rates using specific criteria

Criteria	Partial DOB Cohort: % Linked	Partial DOB Cohort: % Estimated Precision	Full DOB Cohort: % Linked	Full DOB Cohort: % Estimated Precision	Combined Results: % Linked to Spine	Combined Results: *% Linked to CHI*	Combined Results: % Estimated Precision
1. Unique Exact Matches	74.5%	99.76%	89.5%	99.97%	86.1%	*86.0%*	99.93%
2. Original ScotXed "Safe" match criteria	76.2%	99.65%	91.3%	99.87%	87.9%	*87.8%*	99.83%
3. ScotXed "Optimal" Links	94.8%	91.41%	95.3%	98.29%	95.2%	*95.0%*	96.73%
**4. RECOMMENDATION: Calibrated "Optimal" Links: use 1 where Partial DOB; use 3 where Full DOB**					**90.6%**	***90.5%***	**98.57%**
