# Supplementary material for: Data Resource Profile: the Scottish Social Care Survey (SCS) and the Scottish Care Home Census (SCHC)
Source: Int J Popul Data Sci. 2019 Sep 2;4(1):1108. doi: 10.23889/ijpds.v4i1.1108 (PMC8142955; doi:10.23889/ijpds.v4i1.1108)
Supplement: Social Care Survey, NRS Indexing Spine Linkage [file ijpds-04-1108-s002.pdf]

National Records of Scotland

Social Care Survey
NRS Indexing Spine Linkage

20th June 2017

This Document was

Prepared by:	Dave Clark	Indexing Service Manager	20th June 2017
Reviewed by:			
Authorised by:			

Document Distribution

NRS:	Data Linkage and Indexing
Scottish Government:	Health and Social Care Analysis, OCSSA
NSS:	eDRIS Team

Amendment Suggestion

If you have suggested amendments please make them to NRS Indexing Team.

Status Control

Version	Date	Status	Prepared by	Reason for Amendment
0.1	27.06.2017	First draft	Dave Clark	

Contents

1. Introduction 4
2. Data and Methods 4
3. Conclusions and Recommendations 7

1. Introduction

1.1 This report describes the linkage of Social Care Survey (SCS) to the National Records of Scotland (NRS) Research Indexing Spine for the purpose of establishing read-through indexes to be used in linkage projects under the Scottish Informatics and Linkage Collaboration (SILC) model.

1.2 The report describes the quality of the person identifying information (PII) supplied, the methods used to match the PII to the Spine, and the success rate of the matching in terms of linkage rates and crude estimates of the precision (positive predictive value) of the matches.

1.3 Match rates are described in terms of breakdowns of demographic factors derived from the PII, namely Year of Birth, Gender, Deprivation Categories (SIMD 2016 deciles) and Council Area.

1.4 A selection of linkage criteria options were explored and a recommendation is made for the default criteria to be used in linkage projects.

1.5 Full results can be found in the accompanying appendix spreadsheet – "20170627 Linkage Report.xlsx".

2. Data and Methods

2.1 Data from all 32 local authorities were securely transferred to the Indexing Service by ScotXed. Each combination of home postcode, gender and date of birth per encrypted ClientID were provided as separate records, and a code representing Council Area was also included in the dataset. In total, there were 594,380 records in the dataset, pertaining to 488,185 unique ClientID. A person who was represented in more than one local authority would be given a separate ClientID. The coverage of the dataset was anyone on the Home Care Survey (2010-2012), Self-Directed Support Survey (2010-2012) and the Social Care Survey (2013-2016).

2.2 Postcodes from 32,938 (5.5%) of records were either missing or incomplete. Gender could not be determined on 1,225 (0.2%) of records. 3,618 records were missing date of birth and 60 records had date of birth beyond 2017. A disproportionate number of records (3,183 or 0.5%) had year of birth 1899 or 1900. In comparison, a total of only 49 records had year of birth between 1901-1905. More records (57,926 or 9.8%) had a January month of birth than would be expected (50,140 or 8.5%) if there was a Uniform distribution across all months of birth. The biggest data quality issue was the huge amount of records where day of birth was recorded as '01'. There were 186,768 such records, representing 31.6% of the cohort (19,409 or 3.3% would be expected if there was a uniform distribution across days of the year). Looking across council areas, 17 CAs had a disproportionate (>5%) amount of day of birth ='01', including 5 areas where nearly all records had this value in their date of birth. Two council areas also had a disproportionate number of records where day of birth defaulted to '15'.

2.3 The methods used to match the Social Care Survey data to the Spine was as described for linking the pupil census to the historic CHI postcode archive – see http://www.isdscotland.org/Products-and-Services/eDRIS/Docs/20150421-Linking-ScotXed-Data.pdf . Those linkage methods had been further refined to match all combinations of postcode, gender, date of birth at the Scottish Candidate Number (SCN) level against the Spine and this had yielded a linkage rate of 99.1% of SCNs using the ‘optimal’ criteria and 94.9% as unique exact matches. However, the pupil census had much more complete and better quality PII compared to the SCS cohort. Given the data quality issues described in 3.2, much lower linkage results were anticipated. Indeed, a pilot linkage, carried out in 2016 on 2015 SCS data produced a unique exact match rate of 87.4%.

2.4 Given the day of birth issues described in 3.2, the data from the 17 CAs affected and having day of birth recorded as ’01’ or missing, were separated out into a sub-cohort of 175,648 (29.6%) records to be processed differently. The probabilistic linkage involved in this sub-cohort ignored the day of birth completely in scoring the pair comparisons. Thresholds for defining the match categories and definition of ‘optimal’ linkage criteria were also scaled down accordingly.

2.5 Best matches per SCS record were aggregated at the encrypted ClientID level to assign to the SpineID with the best match category. 77.3% of best matches came from SCS records which were processed with full date of birth.

Number of Unique PersonIDs by category of input record from best matching record to Spine			
	- full DOB records	377,171	77.3%
	- partial DOB records	111,014	22.7%
	- total unique PersonIDs	488,185	100.0%

2.6 Crude precision estimates were calculated by applying the match category specific estimates observed in the ScotXed – CHI linkage, to the frequency distribution of matches in this linkage. The workings for this can be seen in the appendix spreadsheet – worksheet “Best match workings”. This shows that while the ScotXed “optimal” match criteria provides the most consistent match rates between records processed with full date of birth (95.3%) and records processed with partial date of birth (94.8%), the estimated precision is much lower in the partial DOB sub-cohort (91.4% vs 98.3%). This is due to the large proportion of best matches which had an equally likely rival SpineID match where day of birth was not included as a matching variable. For example, 10% of these matches were category ‘E’ exact matches, but by definition for this match category, there was an alternative Spine record which would also exact match to that SCS ClientID.

2.7 Given this lack of confidence in the matching ability in the absence of day of birth, our recommendation is that best matches made without this variable be restricted to unique exact matches only. These give a linkage rate of 74.5% for these cases but potentially a precision estimate of 99.78%. Where we do have full date of birth, our recommendation is to use the optimal linkage criteria as per the ScotXed pupil census matching. Taking this “calibrated” approach, the overall combined linkage results in a match rate of 90.6% (442,085 matched records) and a crudely estimated precision of up to 98.6%.

Match rates using specific criteria	Partial DOB Cohort		Full DOB Cohort		Combined Results		
Criteria	% Linked	% Estimated Precision	% Linked	% Estimated Precision	% Linked to Spine	*% Linked to CHI*	% Estimated Precision
1. Unique Exact Matches	74.5%	99.76%	89.5%	99.97%	86.1%	*86.0%*	99.93%
2. Original ScotXed "Safe" match criteria	76.2%	99.65%	91.3%	99.87%	87.9%	*87.8%*	99.83%
3. ScotXed "Optimal" Links	94.8%	91.41%	95.3%	98.29%	95.2%	*95.0%*	96.73%
**4. RECOMMENDATION: Calibrated "Optimal" Links: use 1 where Partial DOB; use 3 where Full DOB**					**90.6%**	***90.5%***	**98.57%**

2.8 The match rates reported above refer to linkage against the Spine. Not every person on the Spine has a CHI number. Encouragingly for the purposes of linkage between Social Care and Health data, there is very little attrition (losing only 520 cases) from the Spine match rate (90.6%) and the CHI match rate (90.5%).

2.9 The Council Areas with the highest match rates are Angus and Dumfries & Galloway (both 98.5%). Clackmannanshire Council had a match rate of just 1%. This was due, not only, to all Dates of Birth being recorded to Year and Month only, but also because Clacks postcodes were truncated to 6 of the full 7 characters. The Council Area with the next lowest match rate was North Lanarkshire (76.7%).

2.10 As noted in section 3.2, there was an over-representation of people born in the year 1899 and 1900, which probably implies that most of these people have an incorrect year of birth. The match rates for these people were accordingly low at 29.3% (1899) and 12.0% (1900). The match rate for people born between 1901 and 1905 was 65.2% and this rate increases up to a percentage in the mid-eighties for people born before 1914. From then on, there is a fairly consistent match rate in the range 87% to 93% for people born between 1914 and 1985. There is a slight decrease in match rate between 1986 and 1991, with a trough of 83.9% in 1988 born people, and then the match rate picks up again. From 2012 births onwards the match rate decreases sharply to just over 50% in 2014. Where there was a missing or invalid (born post-2017) year of birth, only 0.1% of these cases could be linked.

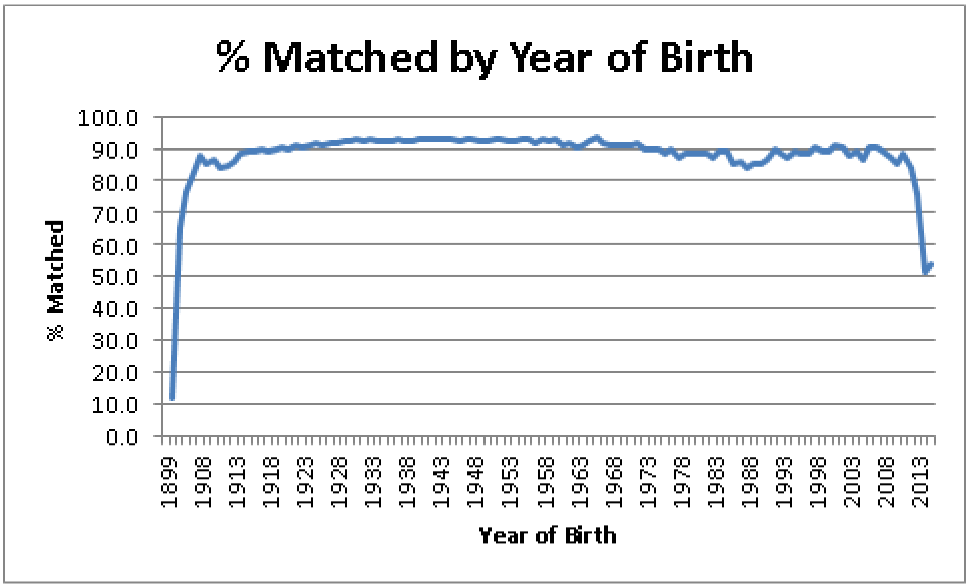

2.11 The match rates among males was 90.4% and was 91.0% in females. None of the 1,186 people with unknown gender could be matched.

2.12 There was no discernible association between match rate and SIMD2016 deciles with people belonging to deciles 2-10 consistently being in the range 93.2% to 93.9%. However, a larger proportion (94.4%) of the most deprived category of people (decile 1) could be successfully matched. Interestingly, this category of area deprivation had the lowest unique exact match rate (87.0%) and there was an obvious trend between lower deprviation and increasing exact matches, peaking at 90.6% in decile 9. Only 19.9% of the people who couldn't be derived a deprivation category could be linked to the spine.

3. Conclusions and Recommendations

3.1 Due to the variability in the quality of the PII provided by different local authorities, 29.6% of records had to be processed separately without day of the month of birth. Our recommended linkage criteria yields a linkage rate, at the council area personID level, of 90.6% to the Spine (90.5% to a CHI number).

3.2 The Indexing Team will hold the read-through to SpineID in a lookup file along with the match categories, linkage weights, full date of birth indicator. This means that researchers can prescribe linkage criteria, other than that recommended in this report, in order to meet the needs of their own research study.

3.3 Data from one specific council area should be explicitly excluded in linkage studies due to a negligible match rate. Linkage rates vary (range 76.7% to 97.9%) across the remaining local authorities and researchers should be aware of these variances when reporting and interpreting results.

3.4 Consideration should be given by SCS data controllers, to obtaining named data for linkage from selected pilot local authorities. This would allow a more robust estimate of precision in this linkage to be calculated.
