# Supplementary material for: Data Resource Profile: the Scottish Social Care Survey (SCS) and the Scottish Care Home Census (SCHC)
Source: Int J Popul Data Sci. 2019 Sep 2;4(1):1108. doi: 10.23889/ijpds.v4i1.1108 (PMC8142955; doi:10.23889/ijpds.v4i1.1108)
Supplement: Care Home Census 2010/11 - 2011/12 Indexing [file ijpds-04-1108-s003.pdf]

**1516-0438**

Care Home Census 2010/11 - 2011/12 Indexing

## Linkage Summary Report

### <u>Stage 1: Preprocessing</u>

| | | | |
|---|---|---|---|
| **Number of Input Records:** | **71,641** | | |
| valid gender | 71,602 | 99.9% | (coded as '1' = male; '2'=female) |
| valid Scottish postcode | 71,641 | 100.0% | |
| valid year of birth | 41,518 | 58.0% | (In range 1902 - 1995) |
| valid month of birth | 41,518 | 58.0% | |
| valid day of birth | 41,518 | 58.0% | |
| - day of birth = '01' | 1,686 | 4.1% | |
| - expected % day of birth = '01' | | 3.3% | |
| filled forename | - | 0.0% | |
| filled surname | - | 0.0% | |
| CHI filled from previous seeding | | 0.0% | |
| **valid gender/postcode/DOB** | **41,496** | **57.9%** | |

| **Number by Census Year** | 2010/11 | 2011/12 |
|---|---|---|
| - Input records | 36,530 | 35,111 |
| - valid gender /postcode /DOB & filled names | 21,918 | 19,578 |
| - % valid | 60.0% | 55.8% |
| - CHI filled from previous seedings | - | - |
| - %CHI filled previously | 0.0% | 0.0% |

**Further pre-processing:**

## Linkage Summary Report

**Stage 2: BigMatch Linkage against the Indexing Spine**

BigMatch is a linkage software program developed and used in-house by the Statistical Research Division, U.S. Bureau of Census. It has been designed to undertake timely matching of very large files (e.g. linking the US census, 300 million x 300 million).

The program is strictly a linkage engine and implements traditional probabilistic record linkage methodology.

The Bigmatch program is designed to extract plausible matches from a large file using several blocking criteria without having to sort the file before each blocking run.

Further details at https://www.census.gov/srd/papers/pdf/rrc2007-01.pdf

In this run, probabilistic weights and match categories similar to those piloted in the pupil census linkage were generated in SAS on the plausible matches generated by BigMatch - see http://www.isdscotland.org/Products-and-Services/eDRIS/Docs/20150421-Linking-ScotXed-Data.pdf

The BigMatch parameters file was set up with the following heirarchical blocking criteria :

| Block number | Block description |
|---|---|
| 0 | Exact matches on Postcode, Sex, DOB |
| 1 | Matches on Postcode & DOB |
| 2 | Matches on 1st 6 characters of Postcode, Sex, DOB |
| 3 | Matches on Postcode, Sex, Year & Month of Birth |
| 4 | Matches on Postcode, Sex, Year & Day of Month of Birth |
| 5 | Matches on Postcode, Sex, Month & Day of Birth |
| 6 | Matches on 1st 5 characters of Postcode, Sex, DOB |
| 7 | Matches on 1st 4 characters of Postcode, Sex, DOB |
| 8 | Matches on Postcode, Sex & Year of Birth |

**Number of pairs above threshold score output from all blocks per batch:**

| Batch Number | CensusID in batch | Number of pairs | Unique CensusID/SpineID combinations above threhold(s) | Unique CensusID above threhold(s) | Unique SpineID above threhold(s) | Unique CensusID/SpineID combinations at best match score |
|---|---|---|---|---|---|---|
| 1 | **71,641** | 141,801 | 69,906 | 39,164 | 44,278 | 40,404 |
| **TOTAL** | **71,641** | **141,801** | **69,906** | **39,164** | **44,278** | **40,404** |

## Stage 3: DEDUPLICATION

**Identify where there are duplicate CensusID across multiple SpineID**

| | |
|---|---|
| Number of CensusID/SpineID combinations at best match score (per CensusID) | **40,404** |
| Number of CensusID matched to single SpineID at best match score | 38,030 |
| Number of unique CensusID | 39,164 |

An automated process is carried out in order to ensure that each CensusID can appear a maximum of only once in the final linked dataset. The CHC dataset contains individuals who appear in more than one census year so multiple CensusID are expected to match to the same SpineID.

| | |
|---|---|
| Step 1: Where CensusID spans>1 SpineID in same block retain lowest ordered SpineID | 39,164 |
| Step 2: Where CensusID spans>1 SpineID in different blocks, drop higher numbered block(s) | 39,164 |
| **Number of census records with best matches to the Spine** | **39,164** |
| **Percentage of census records with best matches to the Spine** | **54.7%** |
| **Final number of census records with best matches after re-categorisation (see Step4)** | **37,752** |
| **Final percentage of census records with best matches after re-categorisation (see Step4)** | **52.7%** |
| **Final number of census records with best matches to health data (CHI number)** | **37,579** |
| **Final percentage of census records with best matches to health data (CHI number)** | **52.5%** |
| **Total Number of Unique Seeded CHI Numbers** | **25,501** |

**1516-0438**

Care Home Census 2010/11 - 2011/12 Indexing

**Linkage Summary Report**

**Stage 4: Match categorisations**

The BigMatch blocking criteria employed in this linkage and the block-specific linkage thresholds were chosen to replicate , as close as possible, the "ScotXed" linkage criteria used for linkages where only Postcode, Sex and Date of Birth are available. The BigMatch score thresholds used in this linkage were set at a value of 9.0 for Block 1 (in order to restrict pairs in this category to where gender is missing) and 5.0 for all other blocks.

The post-BigMatch processing involved re-calculating weights using the ISD Medical Record Linkage scoring system and deriving delta weights representing the distance to the closest rival Spine match.

Broader match categories (compared to the orginal 'ScotXed' criteria) have been assigned to each best match pair and these are defined as follows:

1= Exact Match (including ties).
2= Mis-match on last character of standardised 7-character postcode.
3= Mis-match on one of either Year, Month or Day of Date of Birth.
4= ISD MRL linkage weight >24.0.
5= Lower quality matches.

Further, flags were attached to best matches to create a menu of pre-defined link status as follows:-
Unique **Exact** Matches (broad match category 1 where delta weight > 0);
Original Scotxed **'Safe'** matches (broad match category 1-2 where delta weight >0);
**Optimal** match categories (best balance between precision and recall identified in ScotXed linkage - includes all from broad match categories 1, 2 & 4 and higher quality matches from 3 ).

Matches which did not reach 'Optimal' status were re-defined as non-links.

**Number of initial best matches - by BigMatch Blocking Strategy :-**

| BestBlock | Description | Frequency | Percent |
|---|---|---|---|
| 0 | Exact matches on Postcode, Sex, DOB | 34,083 | 87.0% |
| 1 | Matches on Postcode & DOB | 13 | 0.0% |
| 2 | Matches on 1st 6 characters of Postcode, Sex, DOB | 465 | 1.2% |
| 3 | Matches on Postcode, Sex, Year & Month of Birth | 473 | 1.2% |
| 4 | Matches on Postcode, Sex, Year & Day of Month of Birth | 235 | 0.6% |
| 5 | Matches on Postcode, Sex, Month & Day of Birth | 968 | 2.5% |
| 6 | Matches on 1st 5 characters of Postcode, Sex, DOB | 1,209 | 3.1% |
| 7 | Matches on 1st 4 characters of Postcode, Sex, DOB | 1,508 | 3.9% |
| 8 | Matches on Postcode, Sex & Year of Birth | 210 | 0.5% |
| **Overall** | | 39,164 | 100.0% |

National Records of Scotland

**Number of initial best matches - by Broad match categories :-**

| Broad Category | Description | Frequency | Percent |
|---|---|---|---|
| 1 | Exact Match (including ties). | 34,083 | 87.0% |
| 2 | Mis-match on last character of standardised 7-character postcode. | 925 | 2.4% |
| 3 | Mis-match on one of either Year, Month or Day of Date of Birth. | 1,676 | 4.3% |
| 4 | ISD MRL linkage weight >24.0. | 1,755 | 4.5% |
| 5 | Lower quality matches. | 725 | 1.9% |
| **Overall** | | 39,164 | 100.0% |

**Number of Best Matches by Linkage Criteria**

| | N | % of cohort | Precision* Crude Estimate |
|---|---|---|---|
| **Default - Optimal Links** | **37,752** | **52.7%** | **98.0%** |
| Safe Links | 34,328 | 47.9% | 99.8% |
| Unique Exact Matches | 33,434 | 46.7% | 99.9% |

*Precision estimate based on ScotXed linkages - see http://www.isdscotland.org/Products-and-Services/eDRIS/Docs/20150421-Linking-ScotXed-Data.pdf

- due to different types of population e.g. children living at home versus older people living in communal establishments, these precision estimates should be treated with caution.

**Linkage Summary Report**

## Stage 5: Linkage Rates by Variable Completeness and Demography

| Table of full_details (valid gender/postcode/DOB) by link | link | | | |
|---|---|---|---|---|
| | No | Yes | Total | % Link |
| full_details | | | | |
| No | 30,132 | 13 | 30,145 | **0.0%** |
| Yes | 3,757 | 37,739 | 41,496 | **90.9%** |
| | | | | |
| Total | 33,889 | 37,752 | 71,641 | **52.7%** |

| Table of yob by link | link | | | |
|---|---|---|---|---|
| | No | Yes | Total | % Link |
| yob | | | | |
| | | | | |
| 1900-1910 | 54 | 409 | 463 | **88.3%** |
| 1911 | 25 | 211 | 236 | **89.4%** |
| 1912 | 34 | 296 | 330 | **89.7%** |
| 1913 | 41 | 419 | 460 | **91.1%** |
| 1914 | 58 | 620 | 678 | **91.4%** |
| 1915 | 64 | 678 | 742 | **91.4%** |
| 1916 | 80 | 822 | 902 | **91.1%** |
| 1917 | 88 | 868 | 956 | **90.8%** |
| 1918 | 91 | 1,031 | 1,122 | **91.9%** |
| 1919 | 137 | 1,293 | 1,430 | **90.4%** |
| 1920 | 166 | 1,852 | 2,018 | **91.8%** |

National Records of Scotland

| | | | | |
|---|---|---|---|---|
| 1921 | 167 | 1,877 | 2,044 | **91.8%** |
| 1922 | 149 | 1,838 | 1,987 | **92.5%** |
| 1923 | 146 | 1,824 | 1,970 | **92.6%** |
| 1924 | 162 | 1,814 | 1,976 | **91.8%** |
| 1925 | 153 | 1,658 | 1,811 | **91.6%** |
| 1926 | 169 | 1,684 | 1,853 | **90.9%** |
| 1927 | 148 | 1,537 | 1,685 | **91.2%** |
| 1928 | 130 | 1,375 | 1,505 | **91.4%** |
| 1929 | 130 | 1,398 | 1,528 | **91.5%** |
| 1930 | 126 | 1,285 | 1,411 | **91.1%** |
| 1931 | 107 | 1,128 | 1,235 | **91.3%** |
| 1932 | 112 | 976 | 1,088 | **89.7%** |
| 1933 | 95 | 829 | 924 | **89.7%** |
| 1934 | 77 | 718 | 795 | **90.3%** |
| 1935 | 76 | 617 | 693 | **89.0%** |
| 1936 | 83 | 601 | 684 | **87.9%** |
| 1937 | 37 | 506 | 543 | **93.2%** |
| 1938 | 61 | 554 | 615 | **90.1%** |
| 1939 | 65 | 417 | 482 | **86.5%** |
| 1940 | 43 | 412 | 455 | **90.5%** |
| 1941 | 36 | 343 | 379 | **90.5%** |
| 1942 | 57 | 312 | 369 | **84.6%** |
| 1943 | 49 | 319 | 368 | **86.7%** |
| 1944 | 26 | 283 | 309 | **91.6%** |
| 1945 | 28 | 212 | 240 | **88.3%** |
| 1946 | 27 | 243 | 270 | **90.0%** |

| | | | | |
|---|---|---|---|---|
| 1947 | 22 | 244 | 266 | **91.7%** |
| 1948 | 64 | 228 | 292 | **78.1%** |
| 1949 | 21 | 224 | 245 | **91.4%** |
| 1950 | 22 | 158 | 180 | **87.8%** |
| 1951 | 22 | 163 | 185 | **88.1%** |
| 1952 | 13 | 180 | 193 | **93.3%** |
| 1953 | 29 | 175 | 204 | **85.8%** |
| 1954 | 15 | 130 | 145 | **89.7%** |
| 1955 | 10 | 145 | 155 | **93.5%** |
| 1956 | 13 | 162 | 175 | **92.6%** |
| 1957 | 13 | 143 | 156 | **91.7%** |
| 1958 | 10 | 150 | 160 | **93.8%** |
| 1959 | 19 | 124 | 143 | **86.7%** |
| 1960 | 11 | 156 | 167 | **93.4%** |
| 1961 | 13 | 146 | 159 | **91.8%** |
| 1962 | 12 | 128 | 140 | **91.4%** |
| 1963 | 11 | 133 | 144 | **92.4%** |
| 1964 | 7 | 129 | 136 | **94.9%** |
| 1965 | 6 | 83 | 89 | **93.3%** |
| 1966 | 10 | 99 | 109 | **90.8%** |
| 1967 | 10 | 106 | 116 | **91.4%** |
| 1968 | 4 | 100 | 104 | **96.2%** |
| 1969 | 4 | 95 | 99 | **96.0%** |
| 1970 | 15 | 90 | 105 | **85.7%** |
| 1971-1975 | 13 | 269 | 282 | **95.4%** |
| 1976-1980 | 15 | 244 | 259 | **94.2%** |

| | | | | |
|---|---|---|---|---|
| 1981-1985 | 10 | 223 | 233 | **95.7%** |
| 1986-1990 | 23 | 187 | 210 | **89.0%** |
| post-1990 | 32 | 79 | 111 | **71.2%** |
| Missing | 30,123 | - | 30,123 | **0.0%** |
| | | | | |
| **Total** | 33,889 | 37,752 | 71,641 | **52.7%** |

| Table of simd_decile by link | | | | |
|---|---|---|---|---|
| | link | | | |
| | No | Yes | Total | % Link |
| simd_decile(SIMD 2016 decile) | | | | |
| 1 - most deprived | 2,605 | 3,047 | 5,652 | **53.9%** |
| 2 | 3,765 | 3,672 | 7,437 | **49.4%** |
| 3 | 3,500 | 3,074 | 6,574 | **46.8%** |
| 4 | 2,239 | 2,594 | 4,833 | **53.7%** |
| 5 | 3,635 | 3,979 | 7,614 | **52.3%** |
| 6 | 3,504 | 4,419 | 7,923 | **55.8%** |
| 7 | 3,150 | 4,764 | 7,914 | **60.2%** |
| 8 | 3,470 | 4,162 | 7,632 | **54.5%** |
| 9 | 3,995 | 3,409 | 7,404 | **46.0%** |
| 10 - least deprived | 3,013 | 2,713 | 5,726 | **47.4%** |
| Missing | 1,013 | 1,919 | 2,932 | **65.5%** |
| | | | | |
| **Total** | 33,889 | 37,752 | 71,641 | **52.7%** |

| Table of Sex by link | | | | |
|---|---|---|---|---|
| | link | | | |
| | No | Yes | Total | % Link |
| **Sex** | | | | |
| Male | 10,682 | 11,754 | 22,436 | **52.4%** |
| Female | 23,181 | 25,985 | 49,166 | **52.9%** |
| Missing | 26 | 13 | 39 | **33.3%** |
| | | | | |
| **Total** | 33,889 | 37,752 | 71,641 | **52.7%** |

| Table of Census Year by link | | | | |
|---|---|---|---|---|
| | link | | | |
| | No | Yes | Total | % Link |
| **Census year** | | | | |
| 1011 | 16,690 | 19,840 | 36,530 | **54.3%** |
| 1112 | 17,199 | 17,912 | 35,111 | **51.0%** |
| | | | | |
| **Total** | 33,889 | 37,752 | 71,641 | **52.7%** |

**1516-0438**

Care Home Census 2010/11 - 2011/12 Indexing

**Linkage Summary Report**

**Stage 6: Check number of records per seeded CHI number**

| Number of Care Home Records per Seeded CHI number | Number of CHI | Number of CHC recs |
|---|---|---|
| 1 | 13,883 | 13,883 |
| 2 | 11,242 | 22,484 |
| 3 | 307 | 921 |
| 4 | 61 | 244 |
| 5+ | 8 | 47 |
| Linked to Spine but no CHI number | 173 | 173 |
| Non-Spine matches | 33,889 | 33,889 |
| **Total CHI Matches** | 25,501 | **37,579** |
| **Total Indexed CHC records** | 59,390 | **71,468** |
| **% CHI Matches** | | **52.6%** |

**1516-0438**

Care Home Census 2010/11 - 2011/12 Indexing

**Linkage Summary Report**

**Stage 7: Merge with data supplied by EDRIS - CHI Care Home flag, SMR01, SMR04, SMR50**

eDRIS supplied a file containing CHI numbers and flags of people identified as being in a Care Home in 2010/11 - 2011/12 from SMR01, SMR04, SMR50 & CHI datasets. This was matched using CHI number to the Indexing Spine. The resulting linked SpineIDs were then used to merge with the Care Home Census indexed file. Index numbers representing previously indexed 2012-2016 data have been retained.

| | | |
|---|---|---|
| Number of CHI numbers supplied by eDRIS (2010/11 - 2011/12) | 68,231 | |
| Number and % of eDRIS CHI numbers on Spine | 67,730 | 99.3% |
| | | |
| Number and % of these CHI numbers also in Care Home Census (2010/11 - 2011/12) | 22,373 | 32.8% |
| Number and % of these CHI numbers also in Care Home Census (2010/11 - 2015/16) | 41,288 | 60.5% |
| Number and % of these CHI numbers in previous master file (2012/13 - 2015/16) | 45,369 | 66.5% |
| | | |
| Number and % of CHC CHI numbers not in eDRIS data (2010/11 - 2011/12) | 3,128 | 12.3% |
| | | |
| Number of unseeded CHC records | 34,062 | |
| Number of distinct master indexes amongst unseeded 2010/11 - 2011/12 CHC records | 34,007 | |
| (includes CHC records matched to Spine but no seeded CHI) | | |
| | | |
| Number of records in updated master index file (2010/11 - 2015/16) | 266,151 | |
| Number of records in original master index file (2012/13 - 2015/16) | 178,935 | |
| Number of records in subset master index file (2010/11 - 2011/12) | 87,216 | |
| Number of unique master index numbers (2010/11 - 2015/16) | 169,359 | |
| | | |
| Number of unique Index#1 (2010/11 - 2011/12) to Care Home Census | 71,641 | |
| Number of unique Index#2 (2010/11 - 2011/12) to SPARRA | 71,359 | |
| Number of unique Index#3-7 (2010/11 - 2011/12) to eDRIS | 71,359 | |

?
