# Supplementary material for: Data Resource Profile: the Scottish Social Care Survey (SCS) and the Scottish Care Home Census (SCHC)
Source: Int J Popul Data Sci. 2019 Sep 2;4(1):1108. doi: 10.23889/ijpds.v4i1.1108 (PMC8142955; doi:10.23889/ijpds.v4i1.1108)
Supplement: Care Home Census 2012/13 - 2015/16 Indexing [file ijpds-04-1108-s004.pdf]

1516-0438
Care Home Census 2012/13 - 2015/16 Indexing

National Records of Scotland

Linkage Summary Report

Stage 1: Preprocessing

**Number of Input Records:**	**146,152**		
valid gender	145,938	99.9%	(coded as '1' = male; '2'=female)
valid Scottish postcode	146,152	100.0%	(reformatted into compressed form: e.g. 'G2 1DU' -> 'G21DU')
valid year of birth	138,063	94.5%	(In range 1900 - 2013)
valid month of birth	138,063	94.5%	
valid day of birth	138,063	94.5%	
- day of birth = '01'	5,573	3.8%	
- expected % day of birth = '01'		3.3%	
filled forename	129,878	88.9%	(Includes many instances of initital only)
filled surname	129,943	88.9%	(Includes many instances of initital only)
CHI filled from previous seeding	122,858	84.1%	(10 digits filled)
**valid gender/postcode/DOB & filled names**	**129,670**	**88.7%**	(Including initials only filled names)

**Number by Census Year**	2012/13	2013/14	2014/15	2015/16
- Input records	39,739	36,066	34,916	35,431
- valid gender /postcode /DOB & filled names	30,417	31,560	33,014	34,679
- % valid	76.5%	87.5%	94.6%	97.9%
- CHI filled from previous seedings	29,696	30,610	28,792	33,760
- %CHI filled previously	74.7%	84.9%	82.5%	95.3%

Further pre-processing:
Soundex codes of NYSIIS (following ISD Scotland algorithm) of both Surname and Forename added to reformatted file

Linkage Summary Report

<u>Stage 2: BigMatch Linkage against the Indexing Spine</u>

BigMatch is a linkage software program developed and used in-house by the Statistical Research Division, U.S. Bureau of Census. It has been designed to undertake timely matching of very large files (e.g. linking the US census, 300 million x 300 million).

The program is strictly a linkage engine and implements traditional probabilistic record linkage methodology.

The Bigmatch program is designed to extract plausible matches from a large file using several blocking criteria without having to sort the file before each blocking run.

Further details at https://www.census.gov/srd/papers/pdf/rrc2007-01.pdf

The BigMatch parameters file was set up with the following heirarchical blocking criteria :

Block number	Block description
0	Exact matches on Postcode, Sex, DOB, Full Forename and Surname
1	Matches on Postcode, Sex, DOB, Forename and Surname Initials
2	Matches on 1st 2 characters of Postcode, Sex, DOB, Full Forename and Surname
3	Matches on Sex, DOB, Full Forename and Surname
4	Matches on Postcode, Sex, and DOB
5	Matches on 1st 2 characters of postcode, Sex, Year of Birth, 1st 4 characters of Forename
6	Matches on 1st 2 characters of postcode, Sex, Month and Day of Birth, 1st 4 characters of Forename
7	Matches on 1st 5 characters of Postcode, Sex, Year of Birth, Numeric part of Surname Soundex

Number of pairs above threshold score output from all blocks per batch:

Batch Number	CensusID in batch	Number of pairs	Unique CensusID/SpineID combinations above threhold(s)	Unique CensusID above threhold(s)	Unique SpineID above threhold(s)	Unique CensusID/SpineID combinations at best match score
1	**146,152**	474,598	153,103	131,803	73,341	132,093
**TOTAL**	**146,152**	**474,598**	**153,103**	**131,803**	**73,341**	**132,093**

Stage 3: DEDUPLICATION

Identify where there are duplicate CensusID across multiple SpineID

Number of CensusID/SpineID combinations at best match score (per CensusID)	**132,093**
Number of CensusID matched to single SpineID at best match score	114,653
Number of unique CensusID	131,803

An automated process is carried out in order to ensure that each CensusID can appear a maximum of only once in the final linked dataset. The CHC dataset contains individuals who appear in more than one census year so multiple CensusID are expected to match to the same SpineID.

Step 1: Where CensusID spans>1 SpineID in same block retain lowest ordered SpineID	131,906
Step 2: Where CensusID spans>1 SpineID in different blocks, drop higher numbered block(s)	131,803
**Final number of census records with best matches to the Spine**	**131,803**
**Percentage of census records with best matches to the Spine**	**90.2%**
**Final number of census records with best matches to health data (CHI number)**	**131,501**
**Percentage of census records with best matches to health data (CHI number)**	**90.0%**

1516-0438

Care Home Census 2012/13 - 2015/16 Indexing

Linkage Summary Report

<u>Stage 4: Linkage Quality</u>

The blocking criteria employed in this linkage and the block-specific linkage thresholds were determined iteratively over a number of BigMatch runs by clerically reviewing a limited sample of best match weight pairs per blocking strategy. The final thresholds used in this linkage were set at a value of 5.0 for Blocks 0 through to 4; and a threshold of 10.0 for Blocks 5 through to 7.

After the final BigMatch run and post-run processing , best match pairs were sampled using a stratified random approach. Best match pairs were stratified by the blocking criteria and the integer part of the probabilistic linkage score. Pairs were sorted within each strata by the linkage weight,and a random sample of up to 20 pairs were selected within each block and integer weight.

In total 1,055 pairs were sampled across all strata. Precision estimates were calculated for each strata by dividing the number of good links by the number of pairs in the sample. The expected number of good links per strata were calculated by applying the sample precision estimates to the total number of pairs in the sample. These were then summed over all strata in order to allow precision for the cohort as a whole to be calculated.

Summary Estimate of Precision from Pairs Sampling - by Blocking Strategy :-

BestBlock	Description	Frequency	Percent	Number Sampled	Estimated Precision
0	Exact matches on Postcode, Sex, DOB, Full Forename and Surname	87,769	66.6%	20	100.0%
1	Matches on Postcode, Sex, DOB, Forename and Surname Initials	16,192	12.3%	260	99.8%
2	Matches on 1st 2 characters of Postcode, Sex, DOB, Full Forename and Surname	5,785	4.4%	60	100.0%
3	Matches on Sex, DOB, Full Forename and Surname	703	0.5%	42	98.1%
4	Matches on Postcode, Sex, and DOB	12,218	9.3%	212	86.8%
5	Matches on 1st 2 characters of postcode, Sex, Year of Birth, 1st 4 characters of Forename	6,362	4.8%	159	98.7%
6	Matches on 1st 2 characters of postcode, Sex, Month and Day of Birth, 1st 4 characters of Forename	2,111	1.6%	160	97.8%
7	Matches on 1st 5 characters of Postcode, Sex, Year of Birth, Numeric part of Surname Soundex	663	0.5%	142	95.9%
**Overall**		131,803	100.0%	1,055	

**Precision Estimate**	**98.6%**
**95% CI - Lo**	**98.1%**
**95% CI - Hi**	**99.2%**

National Records of Scotland

Linkage Summary Report

Stage 5: Linkage Rates by Variable Completeness and Demography

Table of full_details (valid gender/postcode/DOB & filled names) by link				
	link			
	No	Yes	Total	% Link
full_details				
No	9,169	7,313	16,482	**44.4%**
Yes	5,180	124,490	129,670	**96.0%**
Total	14,349	131,803	146,152	**90.2%**

Table of yob by link				
	link			
	No	Yes	Total	% Link
yob				
1900-1910	44	346	390	**87.7%**
1911	25	251	276	**90.9%**
1912	53	417	470	**88.7%**
1913	86	661	747	**88.5%**
1914	56	914	970	**94.2%**
1915	63	1,205	1,268	**95.0%**
1916	85	1,497	1,582	**94.6%**
1917	77	1,766	1,843	**95.8%**
1918	76	2,262	2,338	**96.7%**
1919	112	3,083	3,195	**96.5%**
1920	273	4,880	5,153	**94.7%**

**1921**	247	4,880	5,127	**95.2%**
**1922**	201	5,294	5,495	**96.3%**
**1923**	271	6,018	6,289	**95.7%**
**1924**	233	6,089	6,322	**96.3%**
**1925**	267	6,133	6,400	**95.8%**
**1926**	227	6,427	6,654	**96.6%**
**1927**	263	6,002	6,265	**95.8%**
**1928**	251	6,134	6,385	**96.1%**
**1929**	225	5,767	5,992	**96.2%**
**1930**	216	5,683	5,899	**96.3%**
**1931**	237	5,328	5,565	**95.7%**
**1932**	233	4,726	4,959	**95.3%**
**1933**	181	4,032	4,213	**95.7%**
**1934**	157	3,713	3,870	**95.9%**
**1935**	179	3,305	3,484	**94.9%**
**1936**	137	3,122	3,259	**95.8%**
**1937**	126	2,663	2,789	**95.5%**
**1938**	138	2,524	2,662	**94.8%**
**1939**	86	2,076	2,162	**96.0%**
**1940**	124	1,876	2,000	**93.8%**
**1941**	70	1,549	1,619	**95.7%**
**1942**	86	1,489	1,575	**94.5%**
**1943**	65	1,447	1,512	**95.7%**
**1944**	54	1,193	1,247	**95.7%**
**1945**	52	975	1,027	**94.9%**
**1946**	50	991	1,041	**95.2%**

National Records of Scotland

1947	53	1,076	1,129	**95.3%**
1948	74	914	988	**92.5%**
1949	26	792	818	**96.8%**
1950	184	725	909	**79.8%**
1951	25	583	608	**95.9%**
1952	33	631	664	**95.0%**
1953	32	589	621	**94.8%**
1954	37	482	519	**92.9%**
1955	24	464	488	**95.1%**
1956	39	486	525	**92.6%**
1957	23	476	499	**95.4%**
1958	13	443	456	**97.1%**
1959	14	440	454	**96.9%**
1960	17	404	421	**96.0%**
1961	25	413	438	**94.3%**
1962	35	408	443	**92.1%**
1963	21	376	397	**94.7%**
1964	27	389	416	**93.5%**
1965	10	267	277	**96.4%**
1966	16	304	320	**95.0%**
1967	18	330	348	**94.8%**
1968	7	314	321	**97.8%**
1969	11	278	289	**96.2%**
1970	12	290	302	**96.0%**
1971-1975	41	851	892	**95.4%**
1976-1980	40	695	735	**94.6%**

1981-1985	13	670	683	**98.1%**
1986-1990	20	504	524	**96.2%**
post-1990	44	491	535	**91.8%**
Missing	8,089	-	8,089	**0.0%**
Total	14,349	131,803	146,152	**90.2%**

Table of simd_decile by link				
	link			
	No	Yes	Total	**% Link**
simd_decile(SIMD 2016 decile)				
1 - most deprived	1,427	10,548	11,975	**88.1%**
2	1,675	14,432	16,107	**89.6%**
3	1,346	11,703	13,049	**89.7%**
4	847	9,737	10,584	**92.0%**
5	1,917	13,471	15,388	**87.5%**
6	1,222	14,377	15,599	**92.2%**
7	1,501	13,686	15,187	**90.1%**
8	1,447	13,971	15,418	**90.6%**
9	1,254	13,966	15,220	**91.8%**
10 - least deprived	1,220	10,345	11,565	**89.5%**
Missing	493	5,567	6,060	**91.9%**
Total	14,349	131,803	146,152	**90.2%**

National Records of Scotland

Table of Sex by link	link			
	No	Yes	Total	% Link
**Sex**				
**Male**	4,765	42,355	47,120	**89.9%**
**Female**	9,370	89,448	98,818	**90.5%**
**Missing**	214	-	214	**0.0%**
**Total**	14,349	131,803	146,152	**90.2%**

Table of Census Year by link	link			
	No	Yes	Total	% Link
**Census year**				
**1213**	6,945	32,794	39,739	**82.5%**
**1314**	3,567	32,499	36,066	**90.1%**
**1415**	2,239	32,677	34,916	**93.6%**
**1516**	1,598	33,833	35,431	**95.5%**
**Total**	14,349	131,803	146,152	**90.2%**

National Records of Scotland

Linkage Summary Report

Stage 6: Final cleanup

Linked CHI numbers which appeared multiple times were examined in the final linked dataset and a decision process was implemented to reset link status to non-links for CHI numbers where number of matched census records was >8 and link score <14. This resulted in 258 linked census records being re-set as non-matches.

Number of Care Home Records per Seeded CHI number	Freq	Break links	Final Freq
1	26,575	-	26,576
2	32,720	-	32,722
3	32,034	-	32,034
4	35,988	-	35,992
5	3,045	-	3,045
6	564	-	564
7	168	-	168
8	128	-	128
12	12	12	-
35	35	34	-
63	63	61	-
169	169	165	-
14651 (non-CHI matches)	14,651	-	14,923
**Total CHI Matches**	131,501	272	**131,229**
**Total CHC records**	146,152		**146,152**
**% CHI Matches**	90.0%		**89.8%**

Total Number of Unique Seeded CHI Numbers **63,356**

National Records of Scotland

Stage 7: Verification of previous CHI-seeding

CHI-seeded from this Spine linkage	131,229	89.8%
CHI filled from previous seeding	122,858	84.1%
- previous CHI numbers verified	119,579	97.3%
- previous CHI numbers do not match current seeded CHI	874	0.7%
- no current seeded CHI where previous CHI number	2,405	2.0%
CHI seeded where none previously	10,776	7.4%

Linkage Summary Report

Stage 8: Merge with data supplied by EDRIS - CHI Care Home flag, SMR01, SMR04, SMR50

eDRIS supplied a file containing CHI numbers and flags of people identified as being in a Care Home from SMR01, SMR04, SMR50 & CHI datasets. This was matched using CHI number to the Indexing Spine. The resulting linked SpineIDs were then used to merge with the Care Home Census indexed file. A combined flag for "New Admission between 2012/13 to 2015/16" was derived based on the flags provided by both CHC and eDRIS.

Number of CHI numbers supplied by eDRIS	91,747	
Number and % of eDRIS CHI numbers on Spine	91,327	99.5%
Number and % of these CHI numbers also in Care Home Census	58,964	64.3%
Number and % of these CHI numbers not in Care Home census	32,783	35.7%
Number and % of CHC CHI numbers not in eDRIS data	4,392	6.9%
Number of unseeded CHC records	14,923	
Number of distinct indexes amongst unseeded CHC records	14,814	
(includes CHC records matched to Spine but no seeded CHI)		
Number of records in master index file	178,935	
Number of unique master index numbers	110,953	
Number of unique Index#1 to Care Home Census	146,152	
Number of unique Index#2 to SPARRA	96,139	
Number of unique Index#3-7 to eDRIS	96,139	
